# Rickettsial Pathogen Perturbs Tick Circadian Gene to Infect the Vertebrate Host

**DOI:** 10.3390/ijms23073545

**Published:** 2022-03-24

**Authors:** Supreet Khanal, Vikas Taank, John F. Anderson, Hameeda Sultana, Girish Neelakanta

**Affiliations:** 1 Department of Biological Sciences, Old Dominion University, Norfolk, VA 23529, USA; supreet.khanal@fda.hhs.gov (S.K.); vikas.taank@viome.com (V.T.); hsultana@utk.edu (H.S.); 2 Department of Entomology, Connecticut Agricultural Experiment Station, New Haven, CT 06511, USA; john.f.anderson@ct.gov; 3 Center for Molecular Medicine, Old Dominion University, Norfolk, VA 23529, USA

**Keywords:** *Ixodes scapularis*, *Anaplasma phagocytophilum*, ticks, Rickettsial pathogens, CLOCK, circadian oscillation, circadian rhythm, blood feeding, immune regulation

## Abstract

*Ixodes scapularis* is a medically important tick that transmits several microbes to humans, including rickettsial pathogen *Anaplasma phagocytophilum*. In nature, these ticks encounter several abiotic factors including changes in temperature, humidity, and light. Many organisms use endogenously generated circadian pathways to encounter abiotic factors. In this study, we provide evidence for the first time to show that *A. phagocytophilum* modulates the arthropod circadian gene for its transmission to the vertebrate host. We noted a circadian oscillation in the expression of arthropod *clock*, *bmal1*, *period* and *timeless* genes when ticks or tick cells were exposed to alternate 12 h light: 12 h dark conditions. Moreover, *A. phagocytophilum* significantly modulates the oscillation pattern of expression of these genes. In addition, increased levels of *clock* and *bmal1* and decreased expression of Toll and JAK/STAT pathway immune genes such as *pelle* and *jak,* respectively, were noted during *A. phagocytophilum* transmission from ticks to the vertebrate host. RNAi-mediated knockdown of *clock* gene expression in ticks resulted in the reduced expression of *jak* and *pelle* that increased bacterial transmission from ticks to the murine host. Furthermore, *clock*-deficient ticks fed late and had less engorgement weights. These results indicate an important role for circadian modulation of tick gene expression that is critical for arthropod blood feeding and transmission of pathogens from vector to the vertebrate host.

## 1. Introduction

In the Northeastern part of the United States, hard ticks, such as *Ixodes scapularis,* are the primary vectors for several bacterial and viral human pathogens, including the Rickettsial pathogen *Anaplasma phagocytophilum* [1,2]. In nature, the 2-year life cycle of these ticks consists of four phases: eggs; larvae; nymphs; and adults [3]. *Ixodes scapularis*, which is commonly referred as a black-legged tick, is a three-host tick, where larvae, nymphs, and adult stages feed on different hosts [4,5]. Larval or nymphal ticks acquire *A. phagocytophilum* when they feed on an infected reservoir host [6]. Upon entry into ticks, *A. phagocytophilum* is transstadially maintained in their different molting stages [7]. However, evidence on the transovarial transmission for these bacteria is not reported. To survive in the vector, *A. phagocytophilum* modulates various signaling pathways. For example, we previously reported that *A. phagocytophilum* induces expression of the arthropod antifreeze gene (IAFGP) critical for the survival of ticks in the cold [2]. The follow-up study from our group showed that transcription factor Activator Protein-1 (AP-1) induces *iafgp* gene expression [8]. Villar and colleagues reported that the presence of *A. phagocytophilum* leads to overrepresentation of proteins involved in blood digestion and lipid absorption in ticks [9]. Several other recent studies from our and other groups provide strong evidence that *A. phagocytophilum* modulates several signaling pathways in ticks for its survival, colonization, and transmission to the vertebrate host [10,11,12,13,14,15,16,17,18,19].

During its life cycle, *I. scapularis* experiences stress from several abiotic and biotic factors such as temperature, day-length time, light exposure, saturation deficit (an index of humidity), host type and density [20]. The presence of bacterial agents, such as *A. phagocytophilum,* benefits ticks encountering both abiotic and biotic stress [2,9,21]. The geophysical cycles of the Earth results in changes in light and temperature throughout the day and year. These changes could affect the physiology, metabolism, and behavior not only in ticks but also in other organisms, ranging from archaea to humans [22,23]. In 1729 the French Astronomer, Jean Jacques d’ Ortous de Mairan, was the first to indicate that organisms use endogenously generated rhythms to circumvent the pressure created by the geophysical cycles of the Earth [24]. These are termed circadian rhythms in which “circa” refers to “almost” and “dies” refers to “day” in Latin [22,23,24]. The rhythms consist of transcriptional/translational negative feedback loops that cycles close to a period of about 24 h, entrained by light and temperature synchronizers or Zeitgeber, a German name for synchronizer [22,23,24].

There have been extensive investigations from the insect model, *Drosophila melanogaster,* that have revealed important components of the circadian system [25,26,27]. The circadian system is mainly composed of CLOCK (CLK); PERIOD (PER); TIMELESS (TIM); and CYCLE (CYC) [25,26,27]. Aryl hydrocarbon receptor nuclear translocator-like protein 1 (ARNTL) or Brain and Muscle ARNT-Like 1 (BMAL1) is a mammalian ortholog of CYC [28]. Biochemical and genetic evidence has indicated that transcriptional/translational negative feedback loops involve two transcriptional activators (CLK and CYC) and two transcriptional inhibitors (PER and TIM) [29,30,31]. CLK:CYK accumulates in the nucleus, forms a heterodimer and binds to an enhancer box (E-box) containing enhancers upstream of PER and TIM promoters to activate the transcription of later genes [29,30,31]. PER and TIM heterodimerize and translocate to the nucleus to inhibit CLK:CYK, eventually affecting their own transcription [29,30,31]. Increased PER and TIM mRNA levels were noted at the end of the day and the highest levels of PER and TIM protein levels were noted in the second half of the night [29,30,31]. During the day photoreceptor Cryptochrome (CRY) performs light-dependent degradation of TIM [32]. The degradation of TIM results in the destabilization of PER by Double Time (DBT), an ortholog of mammalian casein kinase [33]. The degradation of TIM and destabilization of PER results in re-setting the circadian cycle [32,33]. In addition to binding the E-box regions (CACGTG) of PER and TIM promoters, CLK:CYK can also regulate cyclic expression of several other genes that eventually sets the circadian rhythmicity in various cells and tissues in an organism, thereby altering physiology [29,30,31].

The importance of circadian control on mosquito blood feeding, resistance to dichloro-diphenyl trichloroethane (DDT), permethrin, and transmission of pathogens has been reported [34,35,36,37]. In comparison to the studies that have addressed modulation of circadian pathway by various mosquito or other insect-borne pathogens, no studies have elucidated whether tick-borne pathogens also modulate circadian genes for their survival and transmission from ticks to the vertebrate host. In this study, we screened the *I. scapularis* genome for the presence of circadian genes and report for the first time that the circadian pathway is not only critical for tick blood feeding but also is important for *A. phagocytophilum* transmission from vector to the vertebrate host.

## 2. Results

### 2.1. Identification and Phylogenetic Analysis of Circadian Genes in Ixodes scapularis Genome

**Figure 1:**
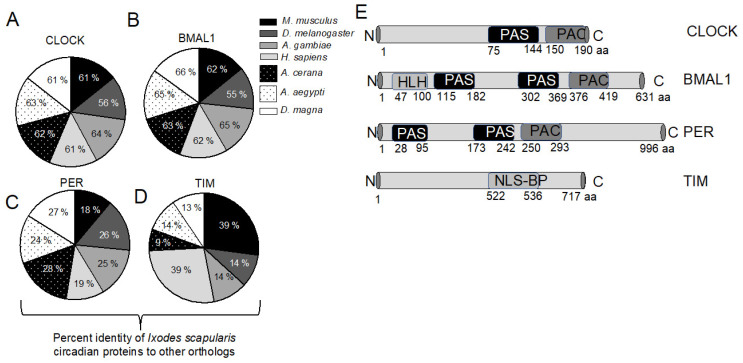
*Ixodes scapularis* circadian proteins share high percent of identity with mouse and human orthologs. (**A**) The pie chart shows the percent identity of *I. scapularis* circadian proteins CLOCK (A); BMAL1 (**B**); PERIOD (**C**); and TIMELESS (**D**) with *M. musculus, D. melanogaster, A. gambiae, H. sapiens, A. cerana, Ae. aegypti* and *D. magna* orthologs. The percent identities were determined using DNASTAR CLUSTALW alignment; (**E**) Domain analyses of *I. scapularis* CLOCK, BMAL1, PERIOD and TIMELESS. PAS indicates a Per-Arnt-Sim (PAS) domain, HLH indicates basic helix-loop-helix domain, NLSBP indicates Bipartite nuclear localization signal and PAC indicates motif C-terminal to PAS domain. Amino acid number corresponding to full length protein and each domain are shown below. N indicates N terminal and C denotes C terminal. Image is not drawn to the scale.

Proteins that play a role in circadian rhythm have been identified in various organisms, including arthropods [26,28,34,38,39]. Using amino acid sequences of circadian proteins from human, mouse, *and Drosophila melanogaster* as a query, blast searches were performed at National Center for Biotechnology Information BLASTP and VectorBase. Tick orthologs of circadian proteins were identified from the *I. scapularis* genome (Supplementary Table S1). The CLUSTALW multiple sequence alignment of CLOCK, BMAL1, PERIOD, and TIMELESS amino acid sequences from *Ixodes scapularis; Homo sapiens; Mus musculus; Drosophila melanogaster; Anopheles gambiae; Aedes aegypti; Apis cerana;* and *Daphnia magna* revealed a high degree of sequence similarity between these orthologs (Supplementary Figure S1). *Ixodes scapularis* CLOCK and BMAL1 shares approximately 60 to 66 % of their identity with orthologs from *M. musculus; An. Gambiae; H. sapiens; A. cerana; Ae. Aegypti;* and *D. magna* and approximately 55 to 56 % identity with *D. melanogaster* orthologs (Figure 1A,B). PERIOD from ticks shares approximately 18 to 19% identity with mouse and human orthologs and 24 to 28% identity with *D. melanogaster; An. Gambiae; A. cerana; Ae. Aegypti;* and *D. magna* (Figure 1C). *Ixodes scapularis* TIMELESS shares approximately 39% identity with both mouse and human orthologs and 9 to 14% identity with *D. melanogaster; An. Gambiae; A. cerana; Ae. Aegypti;* and *D. magna* orthologs (Figure 1D).

**Figure 2:**
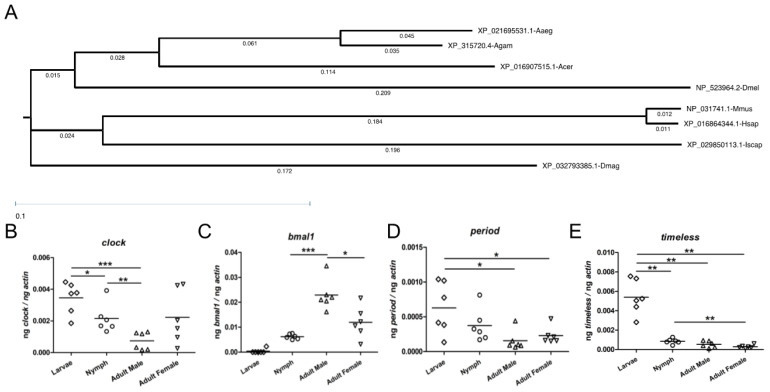
*Ixodes scapularis* circadian genes have varying expression pattern in tick developmental stages. (**A**) Phylogenetic tree showing relatedness of *I. scapularis* CLOCK with *M. musculus*, *D. melanogaster*, *A. gambiae*, *H. sapiens*, *A. cerana*, *Ae. aegypti* and *D. magna* orthologs. The phylogenetic tree was constructed using DNASTAR BIONJ (Neighbor-joining) method. Branch label indicates distance. QRT-PCR analysis showing the levels of *I. scapularis clock* (**B**); *bmal1* (**C**); *period* (**D**) and *timeless* (**E**) transcripts in unfed uninfected larvae (open rhombus), nymphs (open circle), adult male (open triangle) and adult female (open inverted triangle). Levels of circadian gene transcripts were normalized to tick beta-actin levels. Each open and closed circle in nymphs and adults represents transcript levels noted in one tick. However, each circle in larvae denotes a sample generated from three pooled larvae. Statistically, a *p*-value of less than 0.05 was considered significant. * *p* < 0.05, ** *p* < 0.01 and *** *p* < 0.001.

A Per-Arnt-Sim (PAS) domain in circadian proteins has been reported to be an important structural motif that regulates protein-protein interactions in the circadian system [40]. Structural analysis revealed that *I. scapularis* CLOCK contained one, BMAL1 contained two, and PERIOD contained two PAS domains (Figure 1E). A basic helix-loop-helix (HLH) domain that was found in *D. melanogaster* CYCLE and related proteins was also noted to be present in *I. scapularis* BMAL1 (Figure 1E). In addition, Bipartite nuclear localization signal (NLSBP) was noted to be present in *I. scapularis* TIMELESS (Figure 1E). Furthermore, phylogenetic analysis revealed that the *I. scapularis* CLOCK ortholog was close to the clade shared by mouse and human CLOCK proteins (Figure 2A). Mosquito CLOCK proteins fall within the same clade (Figure 2A). *Apis cerana* CLOCK was close to the clade shared by mosquito CLOCK proteins (Figure 2A). *Drosophila melanogaster* CLOCK formed a different clade that was close to mosquitoes and *A. cerana* orthologs, but distant from the mice, human and tick CLOCK proteins (Figure 2A). *Daphnia magna* CLOCK formed a separate clade that was distant from all other CLOCK proteins analyzed in this study (Figure 2A). Similar observations were noted with *I. scapularis BMAL1* (Supplementary Figure S2), PERIOD (Supplementary Figure S3), and TIMELESS (Supplementary Figure S4), where tick proteins were close to the clade formed by mice and human orthologs. These analyses show not only that tick circadian proteins are conserved, but also provide information on the presence of functional domains that are important in circadian pathways.

### 2.2. Tick Circadian Genes Are Developmentally Regulated

As ticks have four phases in their life cycle, we analyzed the expression of *clock, bmal1,* *period and timeless* in all their developmental stages. QRT-PCR analysis was performed to quantify the expression of *clock, bmal1, period and timeless* mRNA levels in unfed uninfected ticks. Expression of these genes were normalized to tick beta-actin levels. QRT-PCR analysis revealed that the expression of *clock* (Figure 2B)*;* *bmal1* (Figure 2C); *period* (Figure 2D); and *timeless* (Figure 2E) was evident in all developmental stages of ticks. All analyzed circadian genes except *bmal1* had significantly (*p* < 0.05) increased expression in larval and nymphal ticks when compared to the expression levels noted in adult ticks (Figure 2B–E). The expression of *bmal1* was noted to be significantly (*p* < 0.05) higher in adult ticks in comparison to levels noted in larvae and nymphs (Figure 2C). Between male and female adult ticks, *bmal1* was noted to be expressed significantly (*p* < 0.05) higher in male ticks in comparison to the female ticks (Figure 2C). No significant differences in the expression of *clock* (Figure 2B); *period* (Figure 2D); and *timeless* (Figure 2E) were evident between male and female ticks. These results indicate the variable expression of circadian genes in different tick developmental stages.

### 2.3. Anaplasma phagocytophilum Perturb Oscillation of Circadian Genes in Ticks and Tick Cells

**Figure 3:**
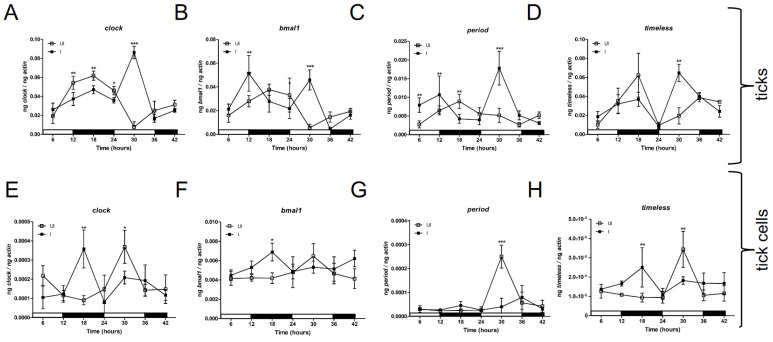
*Anaplasma phagocytophilum* perturbs oscillation of expression of circadian genes in unfed nymphs and tick cells. QRT-PCR analysis showing oscillating expression of circadian genes *clock* (**A**,**E**); *bmal1* (**B**,**F**); *period* (**C**,**G**); and *timeless* (**D**,**H**) in unfed uninfected (open squares) or *A. phagocytophilum*-infected (closed squares) nymphs (**A**–**D**) and tick cells (**E**–**H**) upon exposure to 12 h light: 12 h dark conditions. Uninfected and *A. phagocytophilum*-infected ticks (*n* = 5) and tick cells (*n* = 6) were collected at indicated timepoints. The Y-axis indicates relative mRNA transcripts normalized to tick-beta actin levels and the X-axis represents the time points in hours. Data are presented as the mean ± SD using relative quantification normalized to tick beta-actin mRNA levels. Light and dark phases are indicated as white and black bars, respectively, on the X-axis. Statistical significance between the samples collected at indicated time points was calculated using One way ANOVA analysis and *p* < 0.05 was considered as significant. * *p* < 0.05, ** *p* < 0.01 and *** *p* < 0.001.

The oscillation pattern in gene expression for *clock* and other circadian genes has been observed in several arthropods upon exposure to light: dark conditions [24]. A recent study has shown that ticks respond to both visible and infrared light [41]. As *A. phagocytophilum* modulates various signaling pathways in ticks [8,10,11,12,13,42], we reasoned whether presence of this bacterium has any influence on the expression of the circadian genes. Unfed uninfected and *A. phagocytophilum*-infected nymphs were exposed to alternate 12 h light and 12 h dark conditions for a total period of 42 h (Figure 3). Nymphs were collected at indicated time points and immediately processed for RNA extractions followed by cDNA synthesis, as described [8,11,13]. QRT-PCR analysis showed oscillating expression patterns for all analyzed circadian genes (Figure 3). Uninfected ticks expressed significantly (*p* < 0.05) more of the *clock* transcripts in comparison to *A. phagocytophilum*-infected ticks (Figure 3A) from 12 to 24 h (dark conditions). However, as the light: dark treatment progressed, *A. phagocytophilum*-infected ticks showed significantly (*p* < 0.05) increased *clock* transcripts in comparison to levels noted in uninfected ticks (Figure 3A) at the 30 h time point (light condition). Likewise, the expression of *bmal1, period* and *timeless* was also noted to be significantly (*p* < 0.05) higher in *A. phagocytophilum*-infected ticks in comparison to uninfected ticks in light conditions at the 30 h time point (Figure 3B–D). In addition, the expression of *bmal1* was noted to be significantly (*p* < 0.05) higher in *A. phagocytophilum*-infected ticks in comparison to uninfected ticks at the end of light conditions (12 h). The expression of *period* was noted to be significantly (*p* < 0.05) higher in *A. phagocytophilum*-infected ticks in comparison to the levels noted in uninfected ticks at 6 and 12 h time points (Figure 3B,C).

In tick cells, expression of *clock, bmal1* and *timeless* was noted to be upregulated in the *A. phagocytophilum*-infected group in comparison to the levels noted in uninfected tick cells at 18 h (dark conditions) time points (Figure 3E,F,H). However, no significant upregulation in *period* transcripts were observed in *A. phagocytophilum*-infected tick cells in comparison to the levels noted in uninfected tick cells at all tested time points (Figure 3G). The expression of *clock*, *period* and *timeless* transcripts were upregulated in uninfected tick cells in comparison to the levels noted in *A. phagocytophilum*-infected tick cells at the 30 h time point (light conditions) (Figure 3E,G,H). Taken together, these results show that *A. phagocytophilum* perturbs the oscillation pattern of the expression of *clock, bmal1, period and timeless* genes in ticks and tick cells.

### 2.4. Tick clock and bmal1 Are Upregulated during Transmission of A. phagocytophilum to the Vertebrate Host

**Figure 4:**
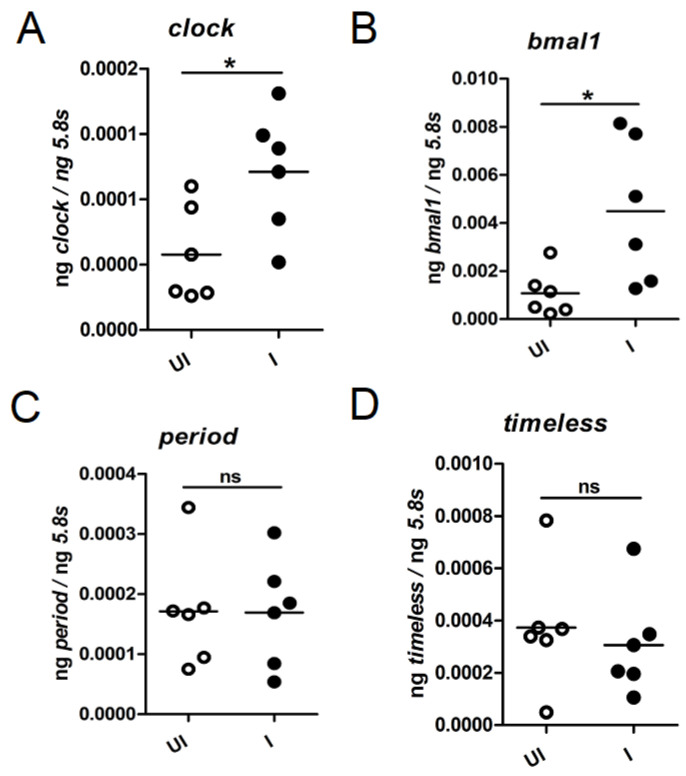
Expression of tick *clock* and *bmal1* are upregulated during transmission of *A. phagocytophilum* from ticks to the murine host. QRT-PCR results showing levels of circadian gene transcripts; *clock* (**A**); *bmal1* (**B**); *period* (**C**); *timeless* (**D**) in uninfected (open circles) or *A. phagocytophilum*-infected (closed circles) nymphs fed on naïve mice. Levels of tick circadian gene transcripts were normalized to tick beta-actin levels. Both uninfected and *A. phagocytophilum*-infected fed ticks were generated from the same batch for consistency. Statistical analysis was performed using Student’s *t*-test comparing uninfected (UI) and *A. phagocytophilum* infected (I) ticks in each group. Each circle represents data from one tick sample. A *p*-value of less than 0.05 was considered significant. * *p* < 0.05, ns—not significant.

As we noted that *A. phagocytophilum* modulates the oscillation pattern of circadian genes, we analyzed whether presence of this bacterium affects the expression of these genes during transmission of this bacterium from ticks to the vertebrate host. Unfed uninfected and *A. phagocytophilum*-infected nymphs were fed on naïve mice. After repletion and upon completion of feeding, ticks were collected and processed for RNA extractions followed by cDNA synthesis. The QRT-PCR analysis indicated that the expression of *clock* and *bmal1* was noted to be significantly (*p* < 0.05) higher in fed *A. phagocytophilum*-infected ticks in comparison to the levels noted in fed uninfected ticks (Figure 4A,B). However, no significant changes in the expression of *period* and *timeless* were noted between fed *A. phagocytophilum*-infected ticks and fed uninfected ticks (Figure 4C,D). No significant changes in the expression of any of the analyzed circadian genes was noted between unfed *A. phagocytophilum*-infected and unfed uninfected ticks that were held in the tick incubator with 14:10 h light: dark cycles for over a two months’ period (Supplementary Figure S5A–D). These results clearly show that expression of *clock* and *bmal1* are specifically upregulated during *A. phagocytophilum* transmission from ticks to the vertebrate host.

### 2.5. Anaplasma phagocytophilum Downregulates Tick Immune Genes during Transmission to the Vertebrate Host

**Figure 5:**
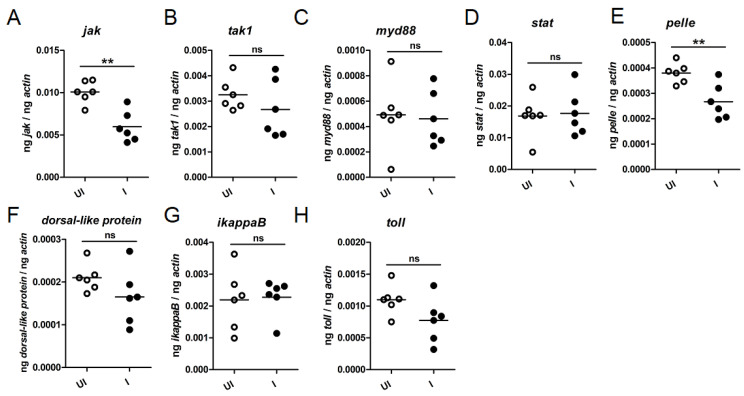
Expression of tick *jak1* and *pelle* are downregulated during transmission of *A. phagocytophilum* from ticks to the murine host. QRT-PCR results showing levels of tick *jak* (**A**); *tak1* (**B**); *myd88* (**C**); *stat* (**D**); *pelle* (**E**); dorsal-like protein (**F**); *ikappaB* (**G**); and *toll* (**H**) transcripts in uninfected (open circles) or *A. phagocytophilum*-infected (closed circles) nymphs fed on naïve mice. Levels of tick immune gene transcripts were normalized to tick-beta actin levels. Statistical analysis was performed using Student’s *t*-test comparing uninfected (UI) and *A. phagocytophilum* infected (I) ticks. Each circle represents data from one tick sample. Statistically significant (*p* < 0.01) comparisons are marked with two asterisks. ns—not significant.

Interactions between circadian components and immune system are well characterized in mammals [43]. However, studies that characterize the role of circadian genes in the immune gene regulation during tick-pathogen interactions are limited. We therefore reasoned whether *A. phagocytophilum* modulates the expression of tick immune genes during transmission from ticks to the vertebrate host. Ticks encode orthologs of several immunity-related genes in their genome [44]. We analyzed expression *of jak; tak1; myd88; stat; pelle; dorsal-like protein; ikappaB;* and *toll* genes in uninfected and *A. phagocytophilum*-infected fed nymphs during transmission of this bacterium from vector to the vertebrate host (Figure 5). QRT-PCR analysis showed that *jak* (Figure 5A) and *pelle* (Figure 5E) transcripts were significantly (*p* < 0.05) reduced in *A. phagocytophilum*-infected fed ticks in comparison to the uninfected fed ticks (Figure 5A,E). No significant differences in the expression of other analyzed tick immune genes were evident during transmission of *A. phagocytophilum* from ticks to the vertebrate host (Figure 5). These results show that *A. phagocytophilum* specifically downregulates certain tick immune genes during its transmission from ticks to the vertebrate host.

### 2.6. RNAi-Mediated Silencing of Clock Expression Affects Tick Blood Feeding

**Figure 6:**
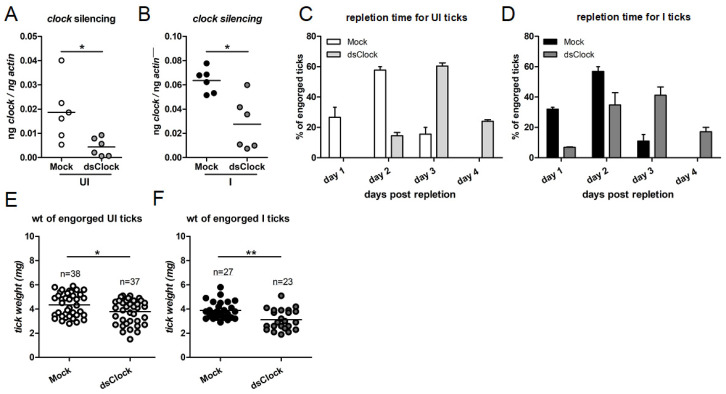
RNAi-mediated silencing of tick *clock* gene expression affects tick engorgement and delays feeding time on the murine host. QRT-PCR analysis showing levels of *clock* transcripts in mock-dsRNA-injected or *clock*-dsRNA-injected uninfected (**A**); or *A. phagocytophilum*-infected (**B**) nymphs fed on naïve mice. Levels of *clock* transcripts were normalized to tick beta-actin levels. Percentage of mock-dsRNA-injected or *clock*-dsRNA-injected uninfected (**C**); or *A. phagocytophilum*-infected (**D**) tick repletion after 72 h post tick placement is shown. Days 1, 2, 3, 4 represents 72, 84, 96 and 108 h post tick placement time points. Engorgement weights of mock-dsRNA-injected or *clock*-dsRNA-injected uninfected (**E**); or *A. phagocytophilum*-infected (**F**) ticks are shown. Each circle represents one tick. Statistical analysis was performed using Student’s *t*-test, and a *p*-value of less than 0.05 was considered significant. * *p* < 0.05, ** *p* < 0.01.

To address whether CLOCK has any role in tick blood feeding, uninfected and *A. phagocytophilum*-infected unfed nymphs were microinjected with mock or *clock-dsRNA*. Microinjection is a technique that can be used to silence tick gene expression and injection of pathogens or inhibitors into ticks [2,8,11,12,13,14,42,45]. The mock- or *clock*-dsRNA-injected uninfected or *A. phagocytophilum*-infected nymphs were fed on naïve mice. Repleted fed ticks were collected and processed for RNA extraction followed by cDNA synthesis and QRT-PCR analysis. The QRT-PCR analysis showed that *clock* transcripts were significantly reduced in *clock*-dsRNA injected uninfected (Figure 6A) or *A. phagocytophilum*-infected nymphs (Figure 6B) in comparison to the respective mock controls (Figure 6A,B). We also noted that *clock*-dsRNA-injected uninfected (Figure 6C) or *A. phagocytophilum*-infected (Figure 6D) ticks feed for longer duration in comparison to the mock-dsRNA-injected control ticks (Figure 6C,D). Furthermore, we also observed that *clock*-dsRNA-injected uninfected (Figure 6E) or *A. phagocytophilum*-infected (Figure 6F) ticks had significantly (*p* < 0.05) less engorgement body weights in comparison to the respective mock-dsRNA injected controls (Figure 6E,F). These results indicate that CLOCK is important for tick blood feeding.

### 2.7. RNAi-Mediated Silencing of clock Expression Affects Immune Gene Expression in Ticks That Facilitates Increased Bacterial Transmission to the Murine Host

**Figure 7:**
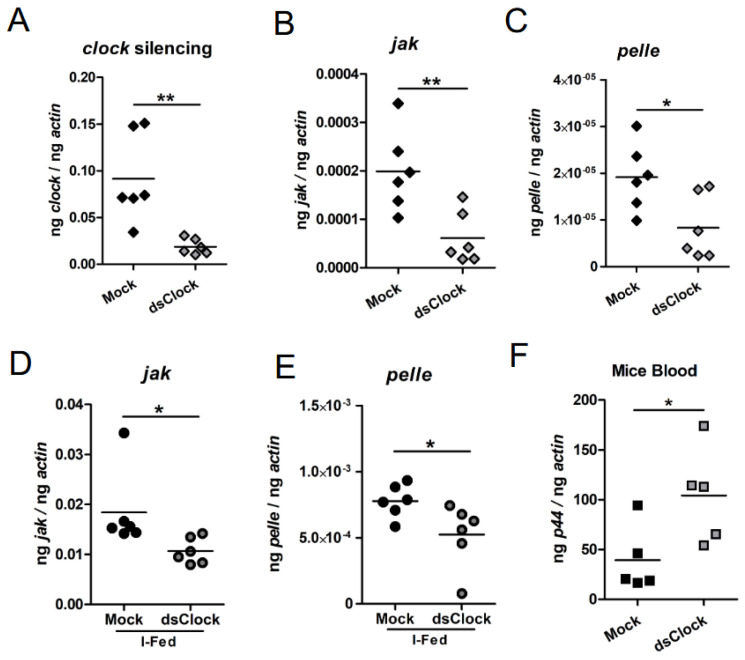
RNAi mediated silencing of arthropod *clock* increases bacterial transmission from ticks to mice. QRT-PCR analysis showing levels of *clock* (**A**); *jak* (**B**); and *pelle* (**C**) transcripts in mock-dsRNA or *clock*-dsRNA treated tick cells. Each rhombus indicates one tick. QRT-PCR analysis showing levels of *jak* (**D**); *pelle* (**E**) transcripts in mock-dsRNA or *clock*-dsRNA-injected ticks. In panels A, B, C, D levels of *clock*, *jak* and *pelle* transcripts were normalized to tick beta-actin levels. (**F**) QRT-PCR analysis showing bacterial burden in murine blood from mice fed with mock-dsRNA-injected or *clock*-dsRNA-injected *A. phagocytophilum*-infected nymphs. Bacterial burden was measured by quantifying amount of *A. phagocytophilum* p44 DNA levels normalized to mouse beta-actin levels. Statistical analysis was performed using Student’s *t*-test, and a *p*-value of less than 0.05 was considered significant. * *p* < 0.05 and ** *p* < 0.01.

To address whether *clock* silencing has any effect in the expression of *jak* and *pelle* transcripts in *I. scapularis* ISE6 in vitro tick cells upon *A. phagocytophilum* infection, these cells were treated with either mock- or *clock*-dsRNA. QRT-PCR analysis showed significantly (*p* < 0.05) less *clock* transcripts in *clock*-dsRNA-treated tick cells in comparison to the levels noted in mock-dsRNA-treated tick cells (Figure 7A). In addition, we noted that *clock*-deficient tick cells had significantly (*p* < 0.05) less *jak* (Figure 7B) and *pelle* (Figure 7C) transcripts in comparison to the levels noted in mock-treated controls. We then microinjected mock- or *clock*-dsRNA into unfed uninfected ticks. QRT-PCR analysis revealed significantly (*p* < 0.05) less *jak* and *pelle* transcripts in *clock*-dsRNA-injected unfed ticks in comparison to levels noted in mock-dsRNA-injected controls (Supplementary Figure S6).

Furthermore, to address whether CLOCK has any role in pathogen transmission, mock or *clock*-dsRNA were injected into *A. phagocytophilum*-infected unfed ticks. These ticks were fed on naive mice. QRT-PCR results revealed silencing of tick *clock* gene expression in *clock*-dsRNA-injected ticks (Figure 7A). Like the observation noted in unfed ticks, significantly less levels of *jak* (Figure 7D) and *pelle* (Figure 7E) transcripts were noted in *clock*-dsRNA-injected *A. phagocytophilum*-infected fed ticks in comparison to the levels noted in mock-dsRNA-injected *A. phagocytophilum*-infected ticks. Furthermore, analysis of the bacterial burden in murine blood showed that mice fed with *clock*-dsRNA-injected *A. phagocytophilum*-infected ticks had significantly more bacterial burden in comparison to the levels noted in blood of mice fed with mock-dsRNA injected *A. phagocytophilum*-infected ticks (Figure 7F). Collectively, these results indicate that tick CLOCK is not only important in regulating immune gene expression but also suggests a role for this molecule during transmission of pathogens from ticks to the vertebrate host.

## 3. Discussion

The molecular mechanisms elucidating the role of circadian genes in various physiological, metabolic, and behavioral pathways have been described in several organisms from different kingdoms [26,38,46,47]. In addition, advances in genetic and molecular tools have enabled researchers to identify and characterize circadian genes in various blood-feeding vectors including mosquitoes, triatomine bugs, and phlebotomine sandflies [24]. Studies that address the importance of the circadian genes in tick blood feeding and interactions with tick-borne pathogens are not reported. Our study provides evidence for the first time that shows the importance of a circadian gene in tick blood-feeding and the transmission of pathogens from a medically important vector. 

The observation of an oscillation pattern for *clock, bmal1, per* and *timeless* gene expression suggests that ticks do respond to light and may possibly modulate circadian gene regulation with the activation of Cry protein. The genome of *I. scapularis* is reported [48]. Ticks encode *cryptochrome* (*cry*) gene in their genome [48]. Tick Cry protein shares approximately 70% identity with other Cry proteins, suggesting a conserved functional role for this protein in circadian gene regulation. Ticks have Haller’s organ, a unique structure found only in ticks and mites on the first tarsus of the front pair of legs [41]. A study provided evidence that Haller’s organ is required for ticks to respond to infrared light [41]. In the same study, the authors indicated that Haller’s organ was not critical for ticks to respond to the visible light. Human visible spectrum typically ranges from 380–750 nm. However, the visible spectrum for these ticks is not known. Some of the studies have shown that most of the Ixodid ticks show one or two peaks in the electroretinogram responses to light (lower peak at 350–380 nm and higher peak around 470–510 nm) [49,50]. Ticks belonging to the *Ixodes* genus have light-sensitive cells on their back [51]. Perret and colleagues have reported the presence of 20–21 cells containing a rhabdomere, located on the dorsolateral side behind coxa 2 in all developmental stages of *Ixodes* ticks [51]. The authors reasoned that these cells may play a role in ticks perception of shifts in light intensity [51]. Collectively, these studies indicate that the arthropod response to light could activate Cry protein that eventually could result in the degradation of TIM protein, thereby regulating the circadian genes in these ticks. 

Several studies have reported that *I. scapularis* becomes more active and feeds later in the day to early morning [52,53,54]. In addition, *I. scapularis* was noted to be also active in the night-time hours [55]. The observation of reduced *jak* and *pelle* transcripts in *clock*-knockdown ticks, in comparison to the levels noted in mock control, suggests that tick CLOCK is positively regulating the expression of *jak1* and *pelle* genes. The observation of significantly reduced *clock* transcripts in unfed *A. phagocytophilum*-infected ticks in comparison to the levels noted in uninfected unfed controls in dark conditions (Figure 3A) and a significant reduction in the levels of *jak* and *pelle* transcripts in *clock*-knockdown *A. phagocytophilum*-infected fed ticks (Figure 7D, E) suggests that this bacterium may be more readily transmissible to the vertebrate host in the night than in the early daytime. This study opens some interesting concepts and novel thoughts for future studies to unravel whether circadian gene regulation is important for ticks to remain active and quest in the night-time, and whether pathogens increase feeding efficiency for ticks during night.

The involvement of circadian control of flight activity, transmission of pathogens, and blood feeding is well documented in mosquitoes [34,35,36,37,56]. The observation of reduced engorgement body weight and delay in blood feeding from *clock*-knockdown ticks provide strong evidence on the role of circadian gene regulation in tick blood feeding. The delay in blood feeding from *clock*-knockdown ticks could be reasoned due to several factors. Some factors could include, but not be limited to, reduced tick mobility and attachment to the host, penetration of tick feeding apparatus into the host skin, delay in cementing tick feeding site, delayed initiation in blood feeding, or inhibition of CLOCK-mediated gene expression (including salivary components) critical for blood feeding. With any or all these factors under circadian control; it would be interesting to elucidate how CLOCK-mediated regulation influences tick blood feeding and pathogen transmission.

Arthropod vectors elicit immune responses by Toll, immune deficiency (IMD), and Janus kinase (JAK)/signaling transducer activator of transcription (STAT pathway) [57]. Several genes that participate in these pathways are noted to be present in the tick genome [44]. A previous study has shown that tick JAK-STAT is important for limiting *A. phagocytophilum* infection in ticks [58]. The tick JAK-STAT pathway was found to be important in regulating antimicrobial peptides [58]. The differential expression pattern for *pelle* transcripts upon *A. phagocytophilum* infection in ticks was reported [17]. In this study, the observation of the downregulation of *jak* and *pelle* transcripts and significantly increased bacterial transmission to the murine host from *clock*-knockdown ticks supports these earlier studies that reported the importance of these genes in tick-*A. phagocytophilum* interactions. 

**Figure 8:**
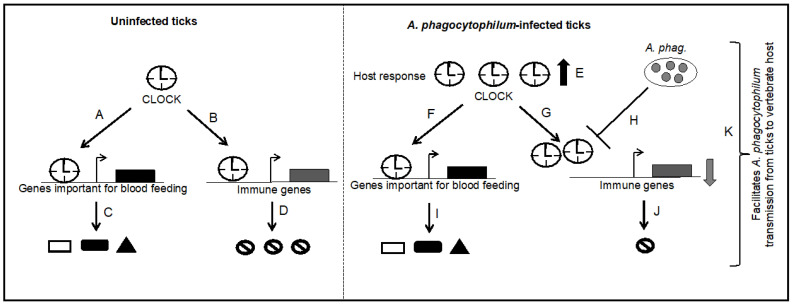
Model showing *A. phagocytophilum*-associated modulation of arthropod circadian gene that facilitates bacterial transmission from ticks to the vertebrate host. In uninfected ticks, CLOCK facilitates transcription of genes critical for blood feeding (**A**); and immune responses (**B**). Translation of these transcripts will lead to the maintenance of endogenous levels of genes important for blood feeding (**C**); and immune responses (**D**). However, in *A. phagocytophilum*-infected ticks, *clock* is upregulated as a part of the host response during transmission of pathogen from ticks to the vertebrate host (**E**). Upregulation of *clock* could result in increased expression of genes critical for blood feeding (**F**); and immune responses (**G**). However, *A. phagocytophilum* suppresses CLOCK-mediated activation of immune genes (**H**); resulting in reduced levels of Jak and Pelle (**J**). *Anaplasma phagocytophilum* may not have any effect on CLOCK-mediated activation of genes involved in blood feeding thereby not affecting tick feeding (**I**); the reduced levels of immune gene expression would facilitate bacterial transmission to the vertebrate host (**K**).

Based on the current findings from this study, we propose a model to elucidate the role of arthropod CLOCK in regulating expression of tick genes involved in blood feeding and immune responses (Figure 8). In uninfected ticks, *clock* is expressed in all developmental stages. CLOCK could directly bind promoters of genes involved in tick blood feeding and immune responses and activate the gene expression (Figure 8). The observation of increased *clock* transcripts and reduced *jak* and *pelle* transcripts during transmission of *A. phagocytophilum* suggests responses from both vector and pathogen. The replication rate for *A. marginale* was noted to be significantly increased in ticks during this bacterial transmission from *Dermacentor andersoni* [59]. Therefore, it is reasonable to hypothesize that during transmission of *A. phagocytophilum* from *I. scapularis* to the murine host, ticks increase *clock* transcripts as a host response to activate *jak* and *pelle* expression, eventually to limit bacterial multiplication (Figure 8). However, *A. phagocytophilum* inhibits CLOCK-mediated regulation to keep *jak* and *pelle* transcripts low for its easy transmission from ticks to the vertebrate host (Figure 8). These observations provide an interesting insight on the molecular changes that happen during transmission of pathogens from ticks to the vertebrate host.

In summary, our study provides evidence on the role of the arthropod *clock* gene in tick blood feeding. In addition, our study noted that the tick-borne rickettsial pathogen could modulate expression of a circadian gene to infect the vertebrate host. Studies like these are novel and are important to understand how pathogens modulate tick gene expression critical for their transmission from vector to the vertebrate host.

## 4. Materials and Methods

### 4.1. Ticks and Bacterial Isolates

*Ixodes scapularis* nymphs were used throughout the study. Various developmental stages of ticks (larvae, nymphs, adult male and female) were either obtained from BEI Resources (NIAID, NIH/CDC) or from Connecticut Agricultural Experiment Station (New Haven, CT, USA) or National Tick Research and Education Resource, Oklahoma State University. All ticks were housed in an environmental incubator (Parameter Generation and Control, Black Mountain, NC, USA) set at 23 ± 2 °C with 94% relative humidity and 14:10 light: dark conditions. *Anaplasma phagocytophilum* NCH-1 strain was used throughout the study and is referred as *A. phagocytophilum*. The NCH-1 strain was maintained in the human promyelocytic cell line (HL-60) as described [8,11]. *Escherichia coli* strains JM109 or DH5alpha were used as cloning hosts.

### 4.2. Mice and Tick Feeding

All tick feeding experiments were performed as described in our previous studies [8,11,13,42]. Briefly, ticks (larvae or nymphs) were fed on C3H/HeN mice (CharlesRiver Laboratories, Wilmington, MA, USA). *Anaplasma phagocytophilum* was maintained in B6.129S7-Rag1tm1Mom/J mice (Jackson Laboratories, Bar Harbor, ME, USA) as described [8,11,13,42]. Infection in mice was performed with *A. phagocytophilum* isolated from in vitro HL-60 cultures as described [8,11,13,42]. During acquisition, naïve larvae or nymphs were fed on uninfected or *A. phagocytophilum*-infected mice. The fed larval ticks were then molted to uninfected or *A. phagocytophilum*-infected unfed nymphs. The molted uninfected or *A. phagocytophilum*-infected unfed nymphs were used in circadian oscillation experiments, gene expression analysis, and transmission experiments. Fed nymphs were used in the expression analysis of circadian genes and measurement of bacterial burden. During transmission, uninfected or *A. phagocytophilum*-infected (including mock or *clock*-dsRNA treated) nymphs were fed on naïve mice. These ticks were then used for expression analysis and measurement of bacterial burden. 

### 4.3. Ethics Statement

All experiments in this study were performed following appropriate guidelines and regulations. All the animal work was carried out in accordance with the Guide for the Care and Use of Laboratory Animals of the National Institute of Health. Experiments in this study were carried out based on the approved animal protocol from Old Dominion University Institutional Animal Care and Use Committee (IACUC) with permit numbers 16-017 and 19-009. Acepromazine was used as a tranquilizer to minimize distress in animals during tick feeding. 

### 4.4. Identification of Circadian Genes from Tick Genome

To identify circadian genes in *Ixodes scapularis* genome, the amino acid sequences from various orthologs were downloaded from National Center for Biotechnology Information (NCBI, http://www.ncbi.nlm.nih.gov/ (accessed on 20 August 2020)) and BLAST searches were performed at VectorBase (www.vectorbase.org (accessed on 20 August 2020)) and NCBI databases. Ortholog sequences were used as a query sequence to conduct BLASTp searches against the *I. scapularis* genome. The top hit sequences were selected based on their lowest E-value using a cutoff E-value of 1 × 10^−30^. SMART and PROSITE algorithms were used to predict domains and motifs in the amino acid sequences. GenBank accession numbers for the sequences analyzed in this study are shown in Supplementary Table S1.

### 4.5. Sequence Alignment and Phylogenetic Analysis

Phylogenetic analysis of circadian proteins was completed using the Neighbor-Joining method in DNASTAR. The neighbor-joining method was applied to construct circadian phylogenetic trees using BIONJ algorithm in DNSTAR. The mRNA or amiono acid sequences of tick circadian proteins were retrieved from the National Center for Biotechnology Information or VectorBase. The amino acid sequences were used for phylogenetic analysis and for prediction of domains.

### 4.6. Circadian Oscillation Experiments

Nymphal ticks and in vitro ISE6 tick cells were used to observe the circadian oscillation of the tick circadian genes. Molted nymphs were housed in the incubator (Parameter Generation and Control, Black Mountain, NC, USA) set at 23 ± 2 °C, 94% relative humidity and 14:10 light: dark conditions. For analysis of circadian genes, all ticks were taken from the same batch of molted nymphs to minimize differences. Five ticks were taken in a clear polypropylene tube and were subjected to alternate 12 h light and 12 h dark conditions in the incubator with similar conditions mentioned above. Independent rounds of experiments were performed using naïve uninfected and *A. phagocytophilum* infected nymphs. For in vitro experiments, 1 × 10^5^ ISE6 tick cells were plated in 12-well cell culture plate. After 24 h, tick cells were infected with cell-free *A. phagocytophilum* as described [8,10,13]. Then after 24 h post infection, cells were subjected to alternative 12 h light and 12 h dark regime. At specific time points during the light: dark cycles’ treatment, both ticks and tick cells were collected in RNA lysis solution (BioRad, Hercules, CA, USA) and further processed for RNA extractions, cDNA synthesis, and gene expression analysis to monitor the oscillation pattern on *clock, timeless, period* and *bmal1*. 

### 4.7. RNA/DNA Extractions and Gene Expression Analysis

RNA and DNA extractions followed by QPCR analysis was performed as described [8,10,11,13]. Briefly, total RNA was extracted using Aurum Total RNA Mini kit (BioRad, Hercules, CA, USA). The extracted RNA from each sample was reverse transcribed into cDNA using iSCRIPT cDNA synthesis kit (BioRad, Hercules, CA, USA). DNA from ticks, tick cells and murine tissues were extracted using DNeasy blood and tissue kit (Qiagen, Germantown, MD, USA). Quantitative Realtime PCR (QRT-PCR) was performed with SYBR green supermix (BioRad, Hercules, CA, USA) using the CFX96 Real-time system (BioRad, Hercules, CA, USA). In QRT-PCR analysis several independent samples were considered, and each cDNA or DNA sample was used in duplicates and average readings from the duplicate samples were considered for the gene expression analysis. The QRTPCR results are shown as the ratio of transcripts from individual genes against the housekeeping genes using relative and absolute quantification methods. Normalization was completed using tick beta-actin or tick 5.8S rRNA transcripts. The real-time expression data were analyzed using Prism 7.0 (GraphPad Prism, San Diego, CA, USA). A student’s *t*-test was used to compare statistical significance between the groups, or ANOVA was used to compare the group’s variations. QRT-PCR analysis was performed on tick circadian genes and several immune genes including *jak; tak1; myd88; pelle; dorsal-like protein; ikappaB; and toll.* Oligonucleotides used for tick *jak; stat;* beta-actin; 5.8S rRNA; and *A. phagocytophilum p44* gene are previously published in our and other studies [8,11,58]. All other primers that were used in this study are listed in Supplementary Table S2.

### 4.8. dsRNA Synthesis and Silencing Experiments in Ticks and Tick Cells

The dsRNA experiments in ticks and tick cells were performed as described [8,10,11,13]. The *clock* gene fragment was amplified using oligonucleotides containing T7 promoter region. The obtained fragment was agarose gel purified using gel purification kit (Qiagen, Germantown, MD, USA) and processed for dsRNA synthesis using MEGAscript RNAi Kit (Ambion Inc., Austin, TX, USA), following the manufacturer’s instructions. The dsRNA generated from multiple cloning site sequence from pL4440 vector was used as a mock control. Microinjections of dsRNA into ticks were performed into the body of ticks and processed as described [8,13]. The microinjected ticks were incubated for 48 h in a desiccator (for recovery) inside an incubator set at 23 ± 2°C, 94% relative humidity and 14/10 h light/dark conditions. After 48 h, microinjected unfed ticks were used for RNA/DNA extractions and processed for gene expression analysis and quantification of bacterial loads. Transfection of dsRNA into tick cells was carried out as described [8,10,11,13]. Briefly, 1 × 10 ^5^ cells were seeded on 12 well plates and incubated for 24 h. Following incubation, tick cells were treated with 750 ng of mock-dsRNA or *clock*-dsRNA mixed with Lipofectamine (Thermoscientific, Waltham, MA, USA). After 6 h, 2X L15-B300 medium was added and plates were incubated for additional 18 h. After 24 h post-dsRNA treatment, *A. phagocytophilum* freshly isolated from infected HL-60 cells was added and cells were incubated for an additional 24 h. After 24 h post-infections, tick cells were collected and processed for RNA or DNA extractions. 

### 4.9. Anaplasma phagocytophilum Transmission Experiments from Ticks to Mice

*Anaplasma phagocytophilum*-infected unfed ticks were microinjected with mock- or *clock*-dsRNA (~4.2 nl/tick). The microinjected ticks were incubated for 24 h for recovery. After 24 h, each group of microinjected ticks was fed on five uninfected mice. The engorged ticks were collected at 72, 96, 108, and 120 h post tick placement, and their tick body weight measurements were recorded immediately. Repleted ticks were cleaned with brushes to remove any mice hair or feces attached to the surface. Weight measurements were taken twice for each tick and average value was considered for the engorgement body weight analysis. In addition, the engorgement and feeding time for repleted ticks were recorded. After five days post-repletion of all engorged ticks, mice were euthanized and blood and tissues such as spleen and liver were collected separately from each group. Total RNA was extracted from the repleted ticks using Aurum Total RNA Mini kit (BioRad, Hercules, CA, USA). The total genomic DNA was extracted from murine tissues using the DNeasy blood and tissue extraction kit (Qiagen, Germantown, MD, USA). QRT-PCR analysis was performed to determine bacterial loads in murine blood and tissues. In addition, QRT-PCR was performed to analyze the expression of *clock* and immune genes in ticks

### 4.10. Statistics

All the data set were statistically analyzed using GraphPad Prism 7 software. The non-paired Student *t*-test was considered to compare the experimental data sets with two variables. One or two-way ANOVA was performed for comparing data obtained from more than two variables. *p* values of <0.05 were considered as significant. Wherever necessary, the statistical method used for the experiments and the *p* values obtained in the data analyses are mentioned in the figures and in legends. In figure panels, *p* < 0.05, *p* < 0.01 and *p* < 0.001 are represented using *, ** and ***, respectively.

## Data Availability

All data are contained either within the main manuscript or in the supporting information document.

## Supplementary Materials

The following supporting information can be downloaded at: https://www.mdpi.com/article/10.3390/ijms23073545/s1.
