# Supplementary material for: Rickettsial Pathogen Perturbs Tick Circadian Gene to Infect the Vertebrate Host
Source: Int J Mol Sci. 2022 Mar 24;23(7):3545. doi: 10.3390/ijms23073545 (PMC8998576; doi:10.3390/ijms23073545)
Supplement: Supplementary file 1 [file ijms-23-03545-s001.zip › ijms-1619925-supplementary.pdf]

International Journal of
Molecular Sciences

MDPI

Supplementary materials

% Identity

% similarity

CLOCK

		A	B	C	D	E	F	G	H
A	NP_031741.1		57.39	63.64	97.73	61.36	63.07	60.23	60.8
B	NP_523964.2	77.27		66.48	56.82	65.91	64.2	59.66	55.68
C	XP_315720.4	73.86	82.95		64.2	79.55	92.05	68.75	63.64
D	XP_016864344.1	98.86	76.7	73.86		61.36	63.64	60.23	60.8
E	XP_016907515.1	73.3	80.11	86.36	73.3		77.27	67.05	61.93
F	XP_021695531.1	72.73	81.82	97.16	72.73	85.8		69.32	63.07
G	XP_032793385.1	70.45	73.3	77.27	70.45	79.55	77.27		61.36
H	XP_029850113.1	71.02	70.45	73.3	70.45	72.73	72.73	71.02	

BMAL1

		A	B	C	D	E	F	G	H
A	NP_031515.1		55.33	60.48	98.97	58.76	60.82	62.2	61.86
B	NP_524168.2	72.85		68.04	55.67	61.51	68.73	62.2	54.98
C	XP_556301.3	76.98	82.82		60.82	78.01	93.81	72.85	64.95
D	NP_001338744.1	98.97	73.2	76.98		58.76	60.82	61.86	61.86
E	XP_016913292.2	72.51	76.63	84.88	72.51		78.35	68.73	63.23
F	AEX32872.1	75.6	84.19	96.22	75.6	85.22		72.51	64.6
G	XP_032781263.1	78.01	79.04	85.22	76.98	80.07	84.54		65.64
H	XP_029845568.1	79.04	75.6	80.07	78.69	76.63	80.07	81.79	

PERIOD

		A	B	C	D	E	F	G	H
A	NP_035196.2		16.28	16.96	51.57	16.96	16.96	16.42	18.47
B	NP_001259194.1	31.46		44.32	18.6	38.3	45.28	25.99	25.72
C	XP_321212.4	31.87	61.01		16.42	39.81	73.6	27.5	24.62
D	NP_002607.2	63.89	31.87	31.6		17.51	16.14	19.43	18.74
E	NP_001315410.1	33.79	51.71	55.54	32.56		39.4	27.22	28.18
F	XP_021710526.1	32.56	60.47	82.63	32.01	56.63		26.4	23.94
G	XP_032778050.1	32.15	42.13	44.73	31.6	43.09	43.78		26.81
H	XP_029823468.1	33.38	42.68	42.41	32.69	44.6	42.82	41.72	

TIMELESS

		A	B	C	D	E	F	G	H
A	NP_001129554.1		17.89	16.89	88.13	8.36	17.39	15.05	38.8
B	NP_001188686.2	34.62		57.69	17.89	7.69	56.69	31.77	13.88
C	XP_001689006.1	34.28	71.24		17.22	7.19	71.91	32.11	14.55
D	NP_003911.2	93.81	35.28	35.45		9.03	17.22	15.38	38.96
E	XP_016914155.2	24.25	22.58	22.41	24.25		8.86	8.86	9.03
F	AAY40757.1	33.11	70.74	80.43	33.95	24.58		31.94	14.38
G	XP_032782117.1	31.44	50.84	50.5	32.61	23.41	50.33		13.55
H	XP_029837764.1	60.2	31.44	31.44	60.03	25.92	32.11	30.6	

Figure S1. Bioinformatic analysis of *Ixodes scapularis* circadian genes. Bioinformatics analysis showing the percent identity (horizontally above black boxed diagonal line) and similarity (vertically below black boxed diagonal line) of the *I. scapularis* CLOCK, BMAL1, PERIOD and TIMELESS with *M. musculus*, *D. melanogaster*, *A. gambiae*, *H. sapiens*, *A. cerana*, *Ae. aegypti* and *D. magna* orthologs. The percent identities were determined using DNASTAR CLUSTALW alignment with the sequences retrieved from NCBI and VectorBase shown in Supplementary Table S1.

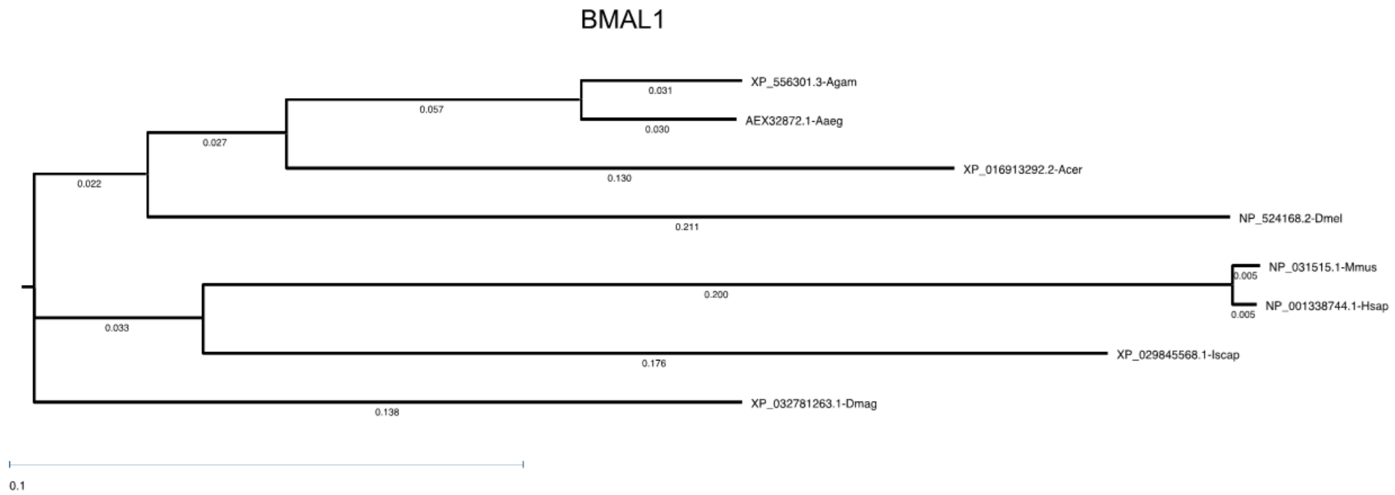

Figure S2. Phylogenetic analysis of *I. scapularis* BMAL1. Phylogenetic tree showing relatedness of *I. scapularis* BMAL1 with *M. musculus, D. melanogaster, A. gambiae, H. sapiens, A. cerana, Ae. aegypti* and *D. magna* orthologs. The phylogenetic tree was constructed using DNASTAR BIONJ (Neighbor-joining) method. Branch label indicates distance.

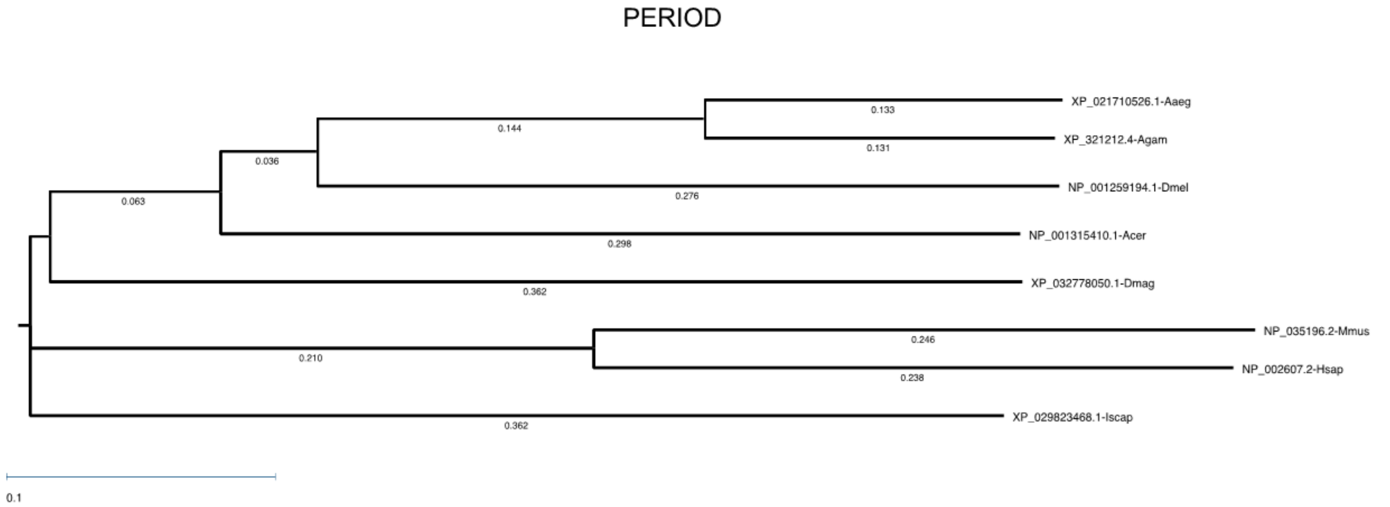

Figure S3. Phylogenetic analysis of *I. scapularis* PERIOD**.** Phylogenetic tree showing relatedness of *I. scapularis* PERIOD with *M. musculus, D. melanogaster, A. gambiae, H. sapiens, A. cerana, Ae. aegypti* and *D. magna* orthologs. The phylogenetic tree was constructed using DNASTAR BIONJ (Neighbor-joining) method. Branch label indicates distance.

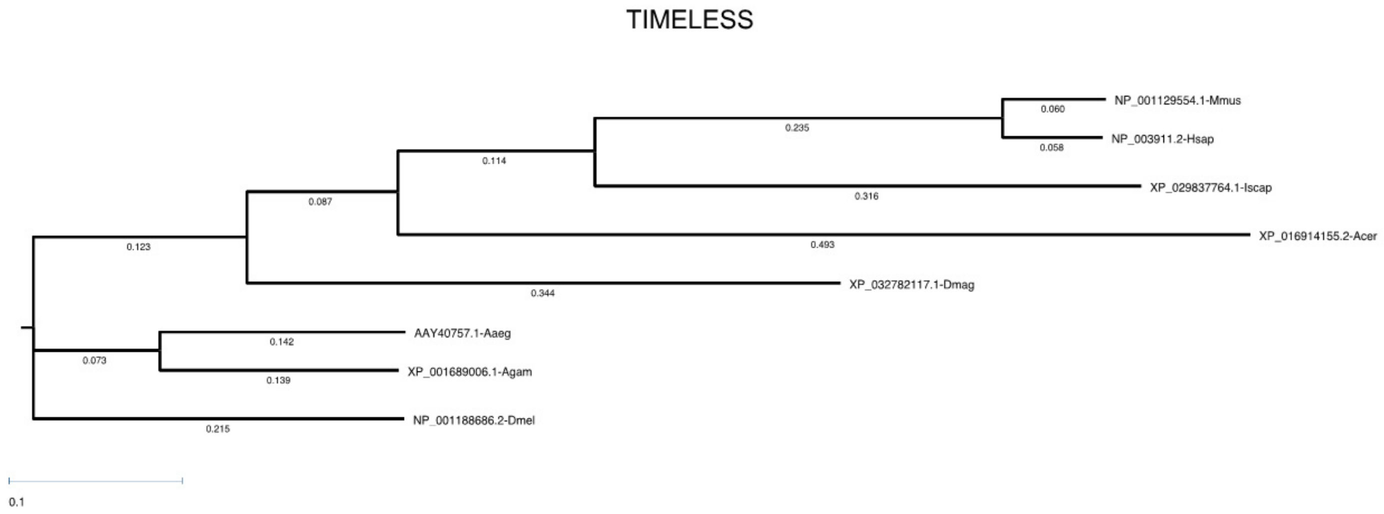

Figure S4. Phylogenetic analysis of *I. scapularis* TIMELESS. Phylogenetic tree showing relatedness of *I. scapularis* TIMELESS with *M. musculus, D. melanogaster, A. gambiae, H. sapiens, A. cerana, Ae. aegypti* and *D. magna* orthologs. The phylogenetic tree was constructed using DNASTAR BIONJ (Neighbor-joining) method. Branch label indicates distance.

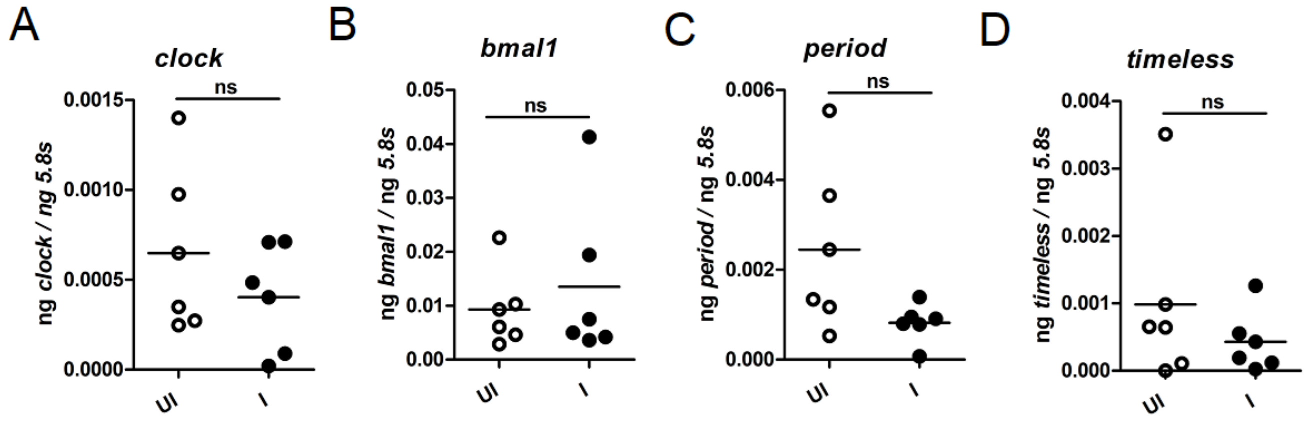

Figure S5. Expression analysis of *I. scapularis* circadian genes in unfed ticks. QRT-PCR results showing levels of circadian gene transcripts; *clock* (**A**), *bmal1* (**B**), *period* (**C**), *timeless* (**D**) in unfed uninfected (open circles) or *A. phagocytophilum*-infected (closed circles) nymphs incubated at 14:10 light:dark conditions for over two months. Levels of tick circadian gene transcripts were normalized to tick beta-actin levels. Both uninfected and *A. phagocytophilum*-infected fed ticks were generated from the same batch for consistency. Statistical analysis was performed using Student's *t*-test. Each circle represent data from one tick sample. *p*-value of less than 0.05 was considered significant. ns—not significant.

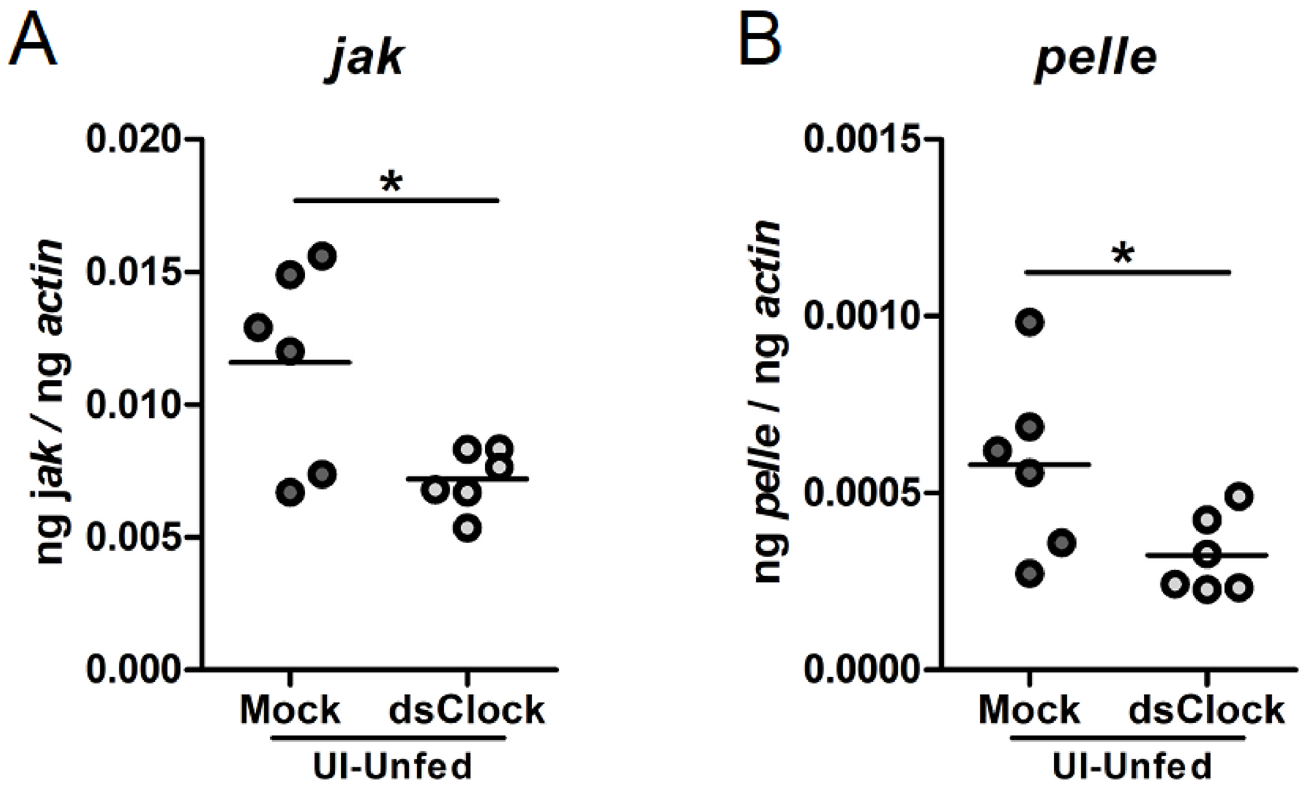

Figure S6. RNAi-mediated silencing of *I. scapularis* clock affects tick *jak* and *pelle* transcripts in unfed ticks. QRT-PCR results showing levels of *I. scapularis jak* (**A**) *or pelle* (***B***) transcripts in mock-treated or *clock*-dsRNA-treated unfed *A. phagocytophilum*-infected ticks. Levels of *jak* and *pelle* transcripts were normalized to tick beta-actin levels. Statistical analysis was performed using Student's *t*-test. Each circle represent data from one tick sample. *p*-value of less than 0.05 was considered significant. * $p < 0.05$.

Table S1. GenBank accession numbers for the proteins analyzed in this study.

Organisms	Proteins			
	CLOCK	BMAL1	PERIOD	TIMELESS
Ixodes scapularis	XP_029850113.1	XP_029845568.1	XP_029823468.1	XP_029837764.1
Homo sapiens	XP_016864344.1	NP_001338744.1	NP_002607.2	NP_003911.2
Mus musculus	NP_031741	NP_031515.1	NP_035196.2	NP_001129554.1
Drosophila melanogaster	NP_523964.2	NP_524168.2	NP_001259194.1	NP_001188686.2
Anopheles gambiae	XP_315720.4	XP_556301.3	XP_321212.4	XP_001689006.1
Aedes aegypti	XP_021695531.1	AEX32872.1	XP_021710526.1	AAY40757.1
Apis cerana	XP_016907515.1	XP_016913292.2	NP_001315410.1	XP_016914155.2
Daphnia magna	XP_032793385.1	XP_032781263.1	XP_032778050.1	XP_032782117.1

Table S2. Oligonucleotides used in this study.

Oligonucleotide Sequence (5'-3')	Gene, Purpose
CGCAAACTGGACTCGGCA	XM_002408003, *Ixodes scapularis bmal1*, QRTPCR
GGCGTCCGCTTCCAAGA	XM_002408003, *Ixodes scapularis bmal1*, QRTPCR
CCGTATCGCTACAAGATGGCA	XM_002410846, *Ixodes scapularis period*, QRTPCR
GCACATCCCTTTGGCGTCT	XM_002410846, *Ixodes scapularis period*, QRTPCR
GCAGCACCTCATCTCCAACTC	XM_002401428, *Ixodes scapularis timeless*, QRTPCR
CCGCCGACGAGTTTGTTCA	XM_002401428, *Ixodes scapularis timeless*, QRTPCR
TAATACGACTCACTATAGGGAGGCAAATAGCCAATGATCGGA	XM_002402163, *Ixodes scapularis clock* with T7 promoter, RNAi
TAATACGACTCACTATAGGGAGCTGGCCGGACCCTGCT	XM_002402163, *Ixodes scapularis clock*, with T7 promoter, RNAi
GCCGGACCCTGCTTCGT	XM_002402163, *Ixodes scapularis clock*, QRTPCR
ACGTGGTAGTAGTCGTATCCTGAAGT	XM_002402163, *Ixodes scapularis clock*, QRTPCR
GGCGGCTGCGTCGGA	Dorsal-like protein, *I. scapularis,* XM_002399338, QRTPCR
CTCGGCGGTGCTGTTGA	Dorsal-like protein , *I. scapularis,* XM_002399338, QRTPCR
CGCAGCAGGCGTTTGTCA	*toll*, *I. scapularis,* XM_002399538, QRTPCR
CGTTCCACTTCACCGATGCT	*toll*, *I. scapularis,* XM_002399538, QRTPCR
GTACCCCGACCTGCCGA	*pelle*, *I. scapularis,* XM_002405411, QRTPCR
CACCGACGACTGTACTCCTCTGA	*pelle*, *I. scapularis,* XM_002405411, QRTPCR
CGACGGCATGATCAGGATGA	*myd88*, *I. scapularis,* XM_002407328, QRTPCR
GGCTGGGGGCACGCT	*myd88*, *I. scapularis,* XM_002407328, QRTPCR
GGGCATGGACCGCAAGT	*ikappaB*, *I. scapularis,* XM_002409625, QRTPCR
GTGGCTAGGTTGGGGCGGT	*ikappaB*, *I. scapularis,* XM_002409625, QRTPCR
CAGATGACCAACAACAAGGGCA	*tak1*, *I. scapularis,* XM_002415486, QRTPCR
CAGTCCTCAAAGGGCTTCCGT	*tak1*, *I. scapularis,* XM_002415486, QRTPCR
